# Proceedings of the American Society of Microscopists

**Published:** 1890-06

**Authors:** 


**Proceedings of the American Society of Microscopists.** Twelfth Annual Meeting. Held at Buffalo, N. Y., August 20, 21, 22, and 23, 1889. Vol. XI. Pp. 182. Committee on Publication, T. J. Burrill, Geo. E. Fell, C. C. Mellor. Buffalo: Bigelow Printing & Publishing Co. 1889.

The proceedings of the twelfth annual meeting of the American Society of Microscopists, published under the direction of the publication committee, attest to the high standard and excellent work done by this great body of scientific men. The Buffalo meeting was considered one of the most successful—not only socially, and in the number of members admitted, but also in the scientific work of the papers presented. Among the more important papers are those by President W. J. Lewis, George E. Blackham, D. S. Kellicott, S. H. Gage and Susanna Phelps Gage, Thomas Taylor, T. J. Burrill, Marshall D. Ewell, George E. Fell, Chevalier Q. Jackson, John M. Stedman, William A. Rogers, George W. Rafter, Lucien Howe, Martin S. Wiard, W. A. Drescher, and H. N. Lyon.

Some excellent plates and wood cuts accompany the papers of D. S. Kellicott, J. M. Stedman, M. S. Wiard and W. A. E. Drescher, adding to the beauty and merit of the same. The society chronicles the death of two of its members the past year, B. L. Oviatt, and Henry Mills, the latter of Buffalo.

The next meeting will be held under the presidency of Dr. George E. Fell, of Buffalo, at Detroit, Mich., beginning August 12, 1890.

